# Supplementary material for: Prior preferences beneficially influence social and non-social learning
Source: Nat Commun. 2017 Oct 10;8:817. doi: 10.1038/s41467-017-00826-8 (PMC5635122; doi:10.1038/s41467-017-00826-8)
Supplement: Supplementary file 1 — Supplementary Information [file 41467_2017_826_MOESM1_ESM.pdf]

Supplementary Figures

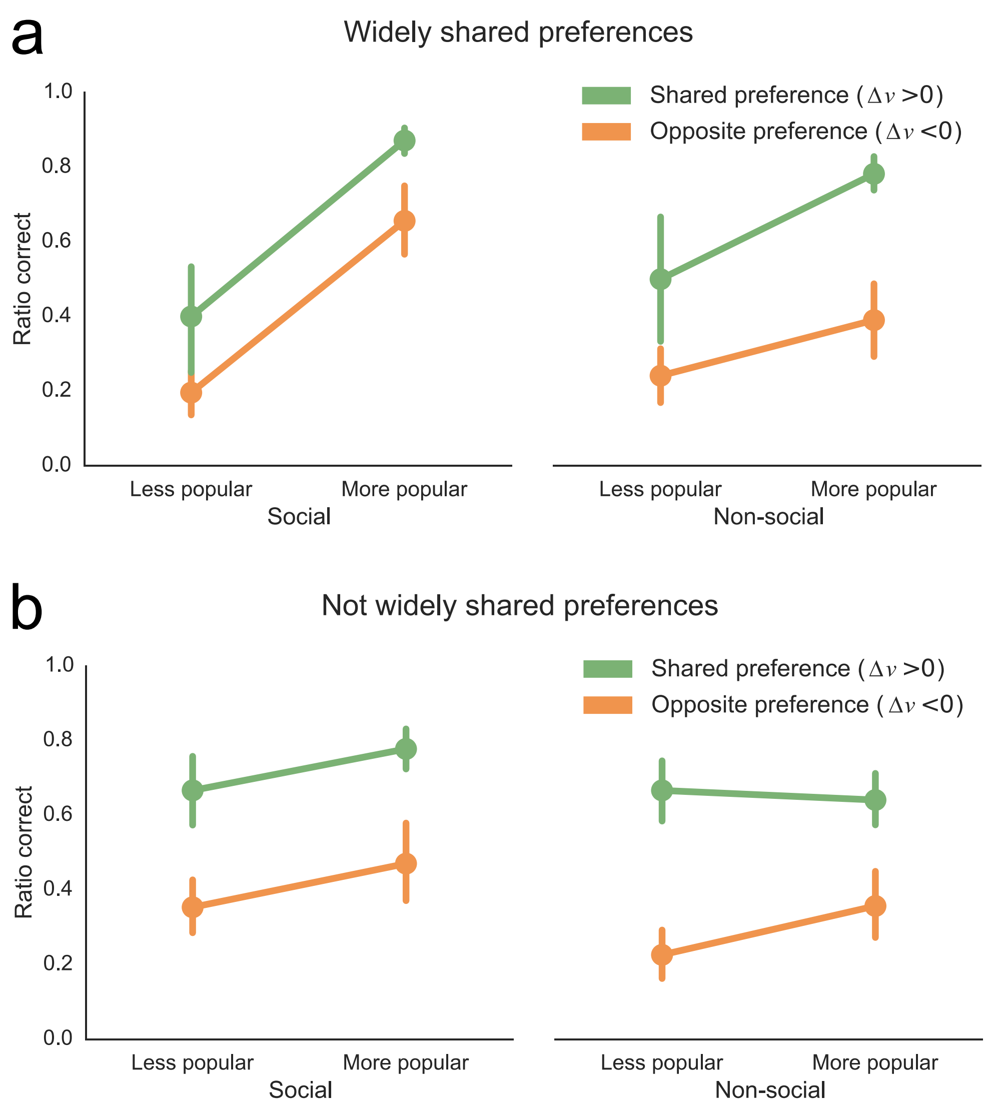

Supplementary Figure 1. Performance on first trials for each item pair, separated by preference congruence and item popularity. **(a)** The effect of item popularity on first trial performance—particularly in the social group—was greater when the popularity of the learned choices was greater (social, shared preference, *n*=217 observations across 31 participants; social, opposite preference, *n*=83 observations; non-social, shared preference, *n*=185 observations across 30 participants; non-social, opposite preference, *n*=99 observations). These data represent the 10 item pairs for which the 61 social and non-social participants showed the most agreement (75-90% bidding more for the same item). **(b)** When preferences were less widely shared, item popularity had less of an effect (social, shared preference, *n*=144 observations; social, opposite preference, *n*=139 observations; non-social, shared preference, *n*=132 observations; non-social, opposite preference, *n*=150 observations). These data represent the 10 item pairs for which the 61 social and non-social participants showed the least agreement in their choices (49-72%). (See main text for statistical tests.) This shows the parametric effect of item popularity illustrated in Fig. 3 (main text). "More popular" items are those preferred by more than 50% of participants in both experiments; "less popular" items were chosen by fewer than 50%. Error bars represent bootstrapped standard errors (ranging between 0.03 and 0.17) clustered by participant.

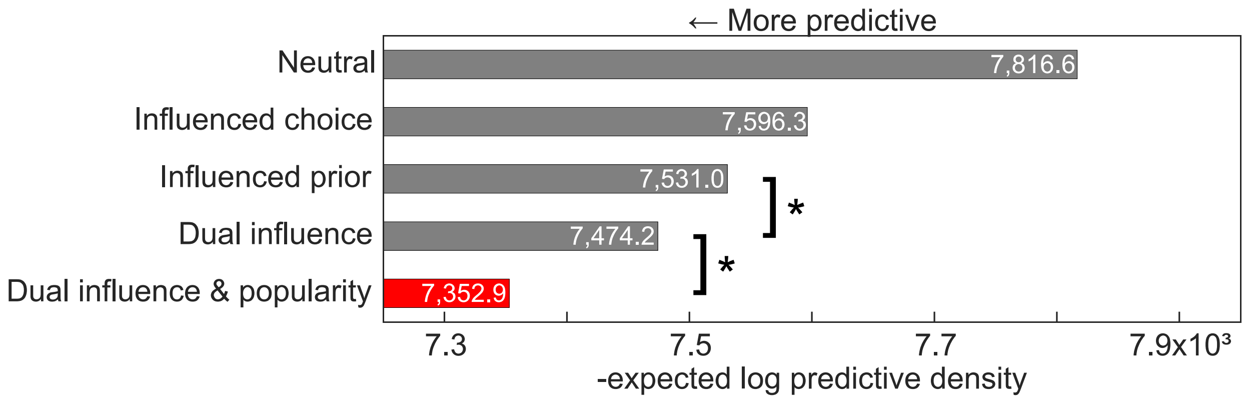

Supplementary Figure 2. Negative estimated log predictive density of the five Rescorla-Wagner-type models fitted to the social group's data (lower means more predictive). * $P<0.05$, two-tailed.

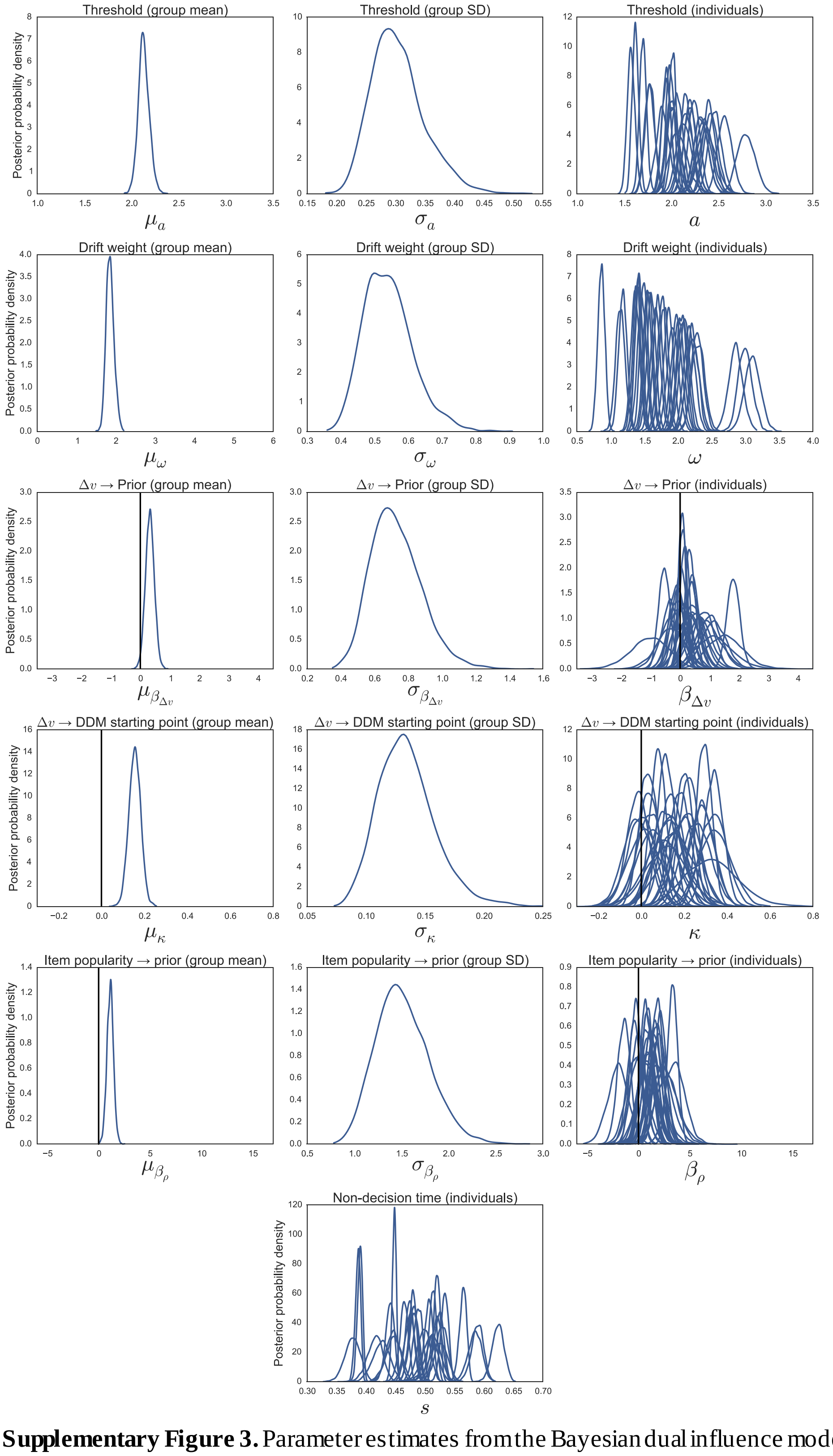

Supplementary Figure 3. Parameter estimates from the Bayesian dual influence model with item popularity fitted to the social group. Left and middle panels indicate estimates of the group means and standard deviations for each parameter (where applicable), and right panels indicate the estimates for each individual participant. Distributions represent uncertainty in the estimates and are kernel density smoothed.

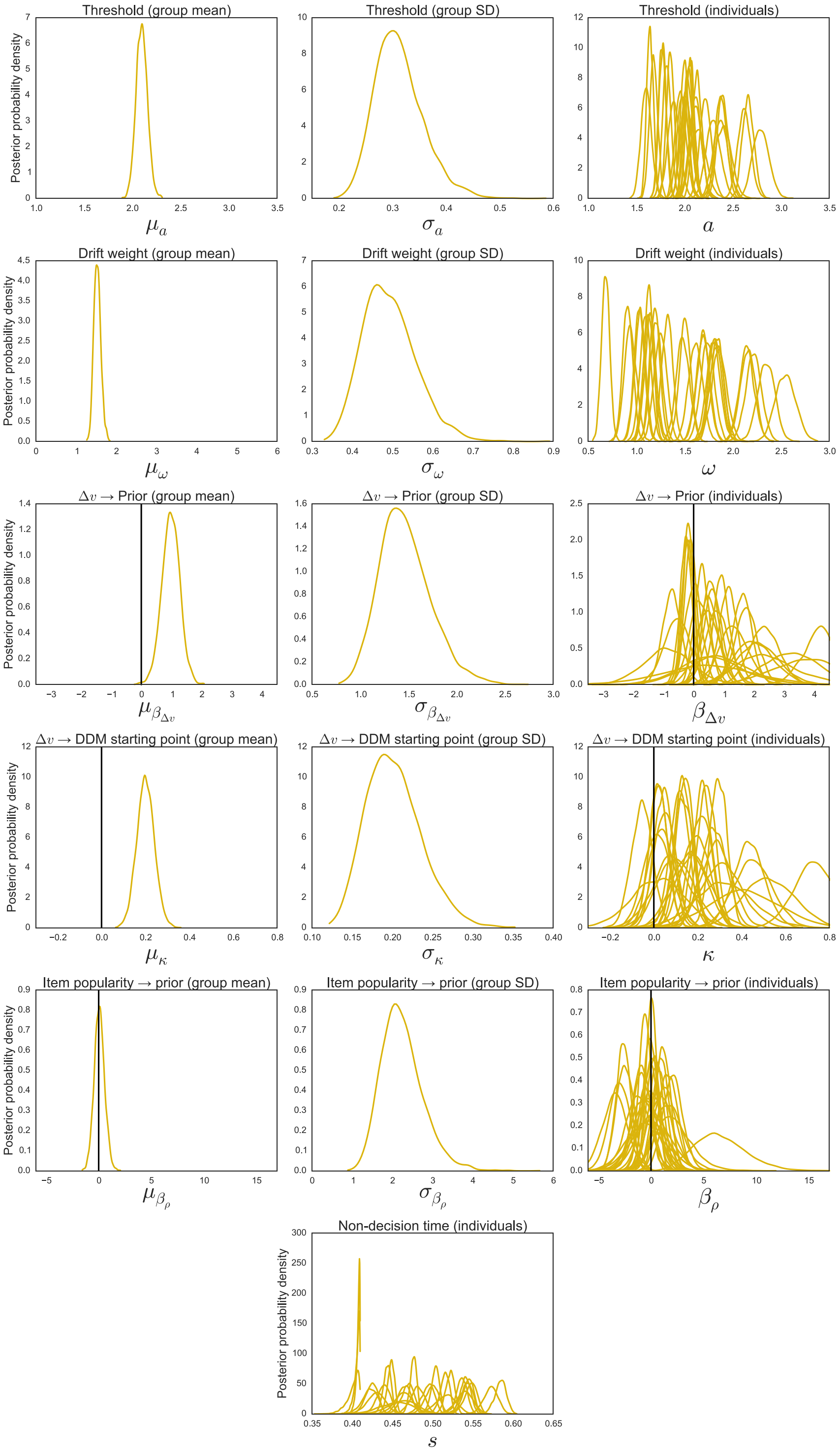

Supplementary Figure 4. Parameter estimates from the Bayesian dual influence model with item popularity fitted to the non-social group. Left and middle panels indicate estimates of the group means and standard deviations for each parameter (where applicable), and right panels indicate the estimates for each individual participant. Distributions represent uncertainty in the estimates and are kernel density smoothed.

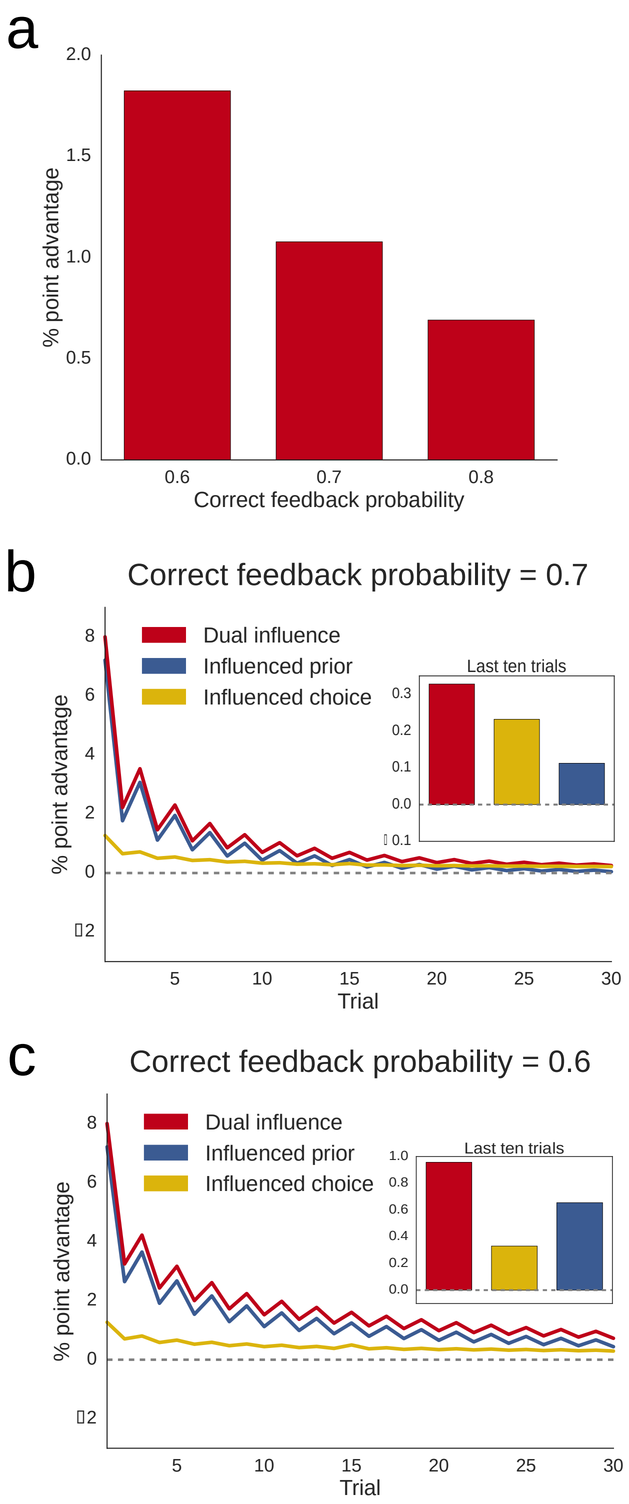

Supplementary Figure 5. Simulated mean performance advantages conferred for different levels of feedback reliability. **(a)** As the uncertainty in the feedback increases—thereby making evidence less reliable—the relative advantage of the dual influence strategy compared to a neutral actor increases. **(b)** When the probability of correct feedback is 0.7, the dual influence strategy confers the greatest advantage over the course of learning. **(c)** When the probability of correct feedback is lowered to 0.6, the advantage conferred by the influenced prior is slower to dissipate, resulting in an even greater overall advantage.

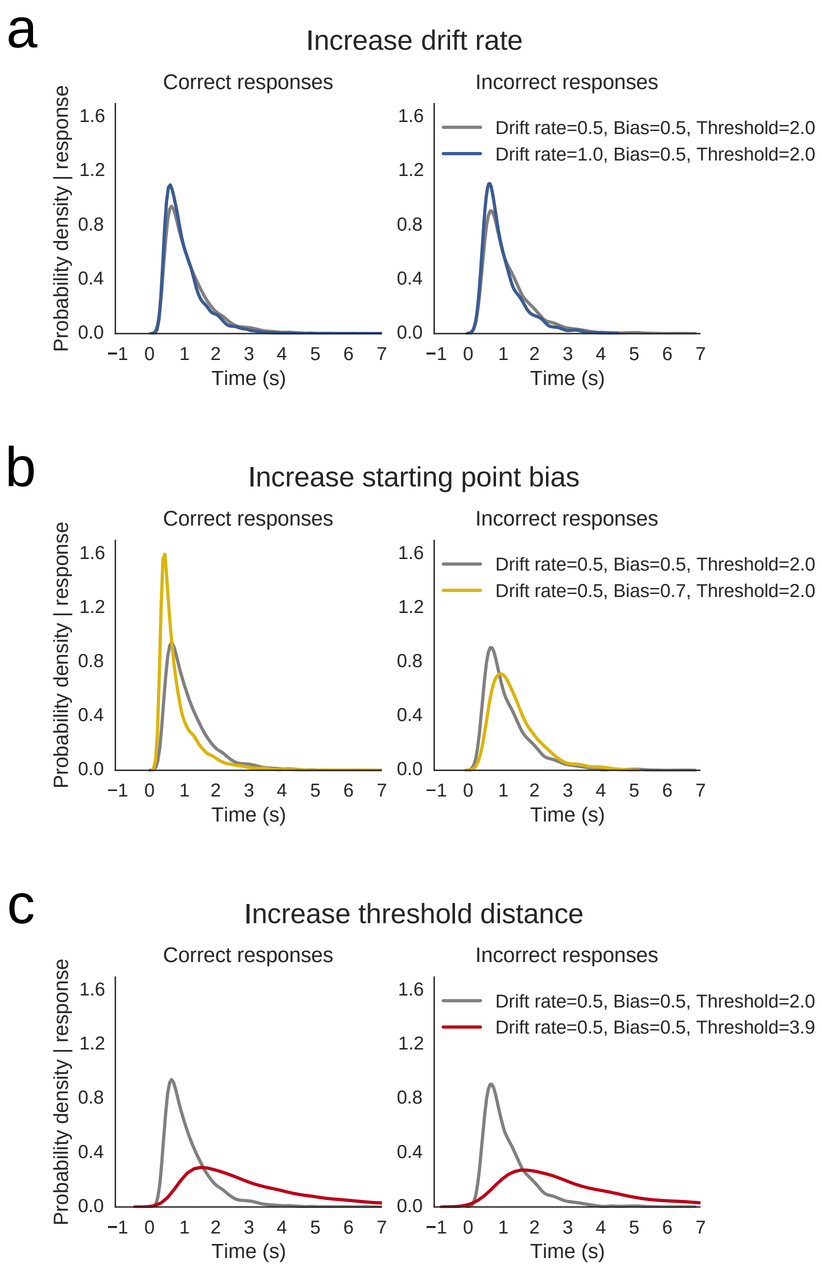

Supplementary Figure 6. Different effects of drift diffusion model parameters on response time distributions. Each of the three models increases accuracy from about 73% (gray plots) to about 88% by changing different parameters. **(a)** Increasing drift rates sharpens response time distributions for both correct and incorrect responses (blue). **(b)** By contrast, biasing the starting point toward the correct response threshold affects correct and incorrect response time distributions differently (yellow). **(c)** Increasing threshold distance broadens response time distributions evenly for correct and incorrect responses (red). For illustration, plots show the probability density of response times given the response (i.e., not weighted by the likelihood of the response itself). Each model is based on 10,000 simulations using [1]. Distributions are kernel density smoothed.

Supplementary Tables

	Variable	Coefficient (SE)	*z* value
**Social, trials 1-30**			
	(Intercept)	1.098*** (0.110)	9.9
	Trial number	0.104*** (0.004)	28.2
	Δv	0.561*** (0.041)	13.9
**Social, trials 1-10**			
	(Intercept)	0.571*** (0.096)	5.9
	Trial number	0.202*** (0.013)	15.5
	Δv	0.563*** (0.053)	10.7
**Social, trials 11-20**			
	(Intercept)	1.478*** (0.351)	4.2
	Trial number	0.111*** (0.020)	5.7
	Δv	0.418*** (0.082)	5.1
**Social, trials 21-30**			
	(Intercept)	3.337*** (0.662)	5.0
	Trial number	0.024 (0.024)	1.0
	Δv	0.826*** (0.099)	8.4

Supplementary Table 1. Correct responses in the social experiment. Models are mixed-effects logistic regressions using bids as the preference congruence measure. Participants are treated as random effects with respect to the intercepts. n=31 participants x 20 item pairs per trial. "Trial number" indicates the number of times the item pair has been presented. "Δv" is the participant's bid for the correct item minus the participant's bid for the incorrect item. * P<0.05, ** P<0.01, *** P<0.001.

	Variable	Coefficient (SE)	*t* value
**Social, trials 1-30**			
LL without Δv: -24952	(Intercept)	0.701 (0.014)	50.1
LL with Δv: -24877***	Trial number	-0.044 (0.001)	-55.9
$\chi^2(1)$=151.02	Δv	-0.117 (0.009)	-12.3
**Social, trials 1-10**			
LL without Δv: -9846	(Intercept)	1.419 (0.041)	34.5
LL with Δv: -9797***	Trial number	-0.157 (0.005)	-30.2
$\chi^2(1)$=96.80	Δv	-0.211 (0.021)	-9.9
**Social, trials 11-20**			
LL without Δv: -7195	(Intercept)	0.217 (0.055)	3.9
LL with Δv: -7170***	Trial number	-0.025 (0.003)	-7.5
$\chi^2(1)$=51.12	Δv	-0.101 (0.014)	-7.2
**Social, trials 21-30**			
LL without Δv: -5768	(Intercept)	-0.098 (0.072)	-1.4
LL with Δv: -5737***	Trial number	-0.008 (0.003)	-2.9
$\chi^2(1)$=61.48	Δv	-0.089 (0.011)	-7.9

Supplementary Table 2. Response times in the social experiment, z-scored by participant. Models are mixed-effects linear regressions using bids as the preference congruence measure. Participants are treated as random effects with respect to the intercepts. Across 31 participants, trials 1-30, n=18,596 observations; trials 1-10, n=6,199 observations; trials 11-20, n=6,198 observations; trials 21-30, n=6,199 observations; response times for four trials were excluded *a priori* due to recording errors. "Trial number" indicates the number of times the item pair has been presented. "Δv" is the participant's bid for the item matching her response minus her bid for its alternative. P values are not noted for coefficients due to ambiguities in their interpretation for mixed-effects linear models. Due to this ambiguity, effects of Δv were evaluated based on whether its inclusion significantly improved the model's fit as measured by a χ^2 test of their log-likelihoods (LL). * P<0.05, ** P<0.01, *** P<0.001.

	Variable	Coefficient (SE)	*z* value
**Social, trials 1-30**			
	(Intercept)	1.105*** (0.113)	9.8
	Trial number	0.105*** (0.004)	28.3
	Same choice	0.492*** (0.029)	17.0
**Social, trials 1-10**			
	(Intercept)	0.563*** (0.099)	5.7
	Trial number	0.206*** (0.013)	15.6
	Same choice	0.560*** (0.038)	14.9
**Social, trials 11-20**			
	(Intercept)	1.481*** (0.353)	4.2
	Trial number	0.111*** (0.020)	5.7
	Same choice	0.362*** (0.058)	6.2
**Social, trials 21-30**			
	(Intercept)	3.348*** (0.660)	5.1
	Trial number	0.024 (0.024)	1.0
	Same choice	0.491*** (0.074)	6.6

Supplementary Table 3. Correct responses in the social experiment. Models are mixed-effects logistic regressions using choices as the preference congruence measure. Participants are treated as random effects with respect to the intercepts. *n*=31 participants x 20 item pairs per trial. “Trial number” indicates the number of times the item pair has been presented. “Same choice” is whether the participant consistently chose the same item as her partner (same=1, other=-1); because participants indicated choices between each item pair twice, inconsistent choices were coded as 0. * P<0.05, ** P <0.01, *** P <0.001.

	Variable	Coefficient (SE)	*t* value
**Social, trials 1-30**			
LL without choice: -24952	(Intercept)	0.702 (0.014)	49.9
LL with choice: -24892***	Trial number	-0.044 (0.001)	-56.1
$\chi^2(1)$=120.42	Same choice	-0.078 (0.007)	-11.0
**Social, trials 1-10**			
LL without choice: -9846	(Intercept)	1.425 (0.041)	34.8
LL with choice: -9801***	Trial number	-0.157 (0.005)	-30.3
$\chi^2(1)$=89.36	Same choice	-0.153 (0.016)	-9.5
**Social, trials 11-20**			
LL without choice: -7195	(Intercept)	0.217 (0.055)	3.9
LL with choice: -7181***	Trial number	-0.026 (0.003)	-7.6
$\chi^2(1)$=28.01	Same choice	-0.055 (0.010)	-5.3
**Social, trials 21-30**			
LL without choice: -5768	(Intercept)	-0.100 (0.072)	-1.4
LL with choice: -5743***	Trial number	-0.008 (0.003)	-2.9
$\chi^2(1)$=50.88	Same choice	-0.060 (0.008)	-7.2

Supplementary Table 4. Response times in the social experiment, z-scored by participant. Models are mixed-effects linear regressions using choices as the preference congruence measure. Participants are treated as random effects with respect to the intercepts. Across 31 participants, trials 1-30, *n*=18,596 observations; trials 1-10, *n*=6,199 observations; trials 11-20, *n*=6,198 observations; trials 21-30, *n*=6,199 observations; response times for four trials were excluded *a priori* due to recording errors. “Trial number” indicates the number of times the item pair has been presented. “Same choice” is whether the participant’s response matched her preference during the choice task (matched=1, did not match=-1); because participants indicated choices between each item pair twice, inconsistent choices were coded as 0. *P* values are not noted for coefficients due to ambiguities in their interpretation for mixed-effects linear models. Due to this ambiguity, effects of the “same choice” variable were evaluated based on whether its inclusion significantly improved the model’s fit as measured by a χ^2 test of their log-likelihoods (LL). * P<0.05, ** P<0.01, *** P<0.001.

	Variable	Coefficient (SE)	*z* value
**Social, trials 2-30**			
	(Intercept)	0.649*** (0.137)	4.7
	Trial number	0.093*** (0.004)	23.5
	Last feedback	0.986*** (0.062)	15.8
	Gap	-0.003 (0.002)	-1.5
	Δv	0.486*** (0.043)	11.3
**Social, trials 2-10**			
	(Intercept)	-0.128 (0.146)	-0.9
	Trial number	0.170*** (0.016)	10.6
	Last feedback	1.413*** (0.085)	16.7
	Gap	-0.004 (0.003)	-1.7
	Δv	0.430*** (0.059)	7.3
**Social, trials 11-20**			
	(Intercept)	1.232** (0.380)	3.2
	Trial number	0.107*** (0.020)	5.4
	Last feedback	0.495*** (0.124)	4.0
	Gap	-0.003 (0.003)	-1.0
	Δv	0.415*** (0.082)	5.1
**Social, trials 21-30**			
	(Intercept)	3.062*** (0.692)	4.4
	Trial number	0.022 (0.024)	0.9
	Last feedback	0.378* (0.160)	2.4
	Gap	0.001 (0.004)	0.2
	Δv	0.827*** (0.099)	8.3

Supplementary Table 5. Correct responses in the social experiment with additional controls. Models are mixed-effects logistic regressions using bids as the preference congruence measure and controlling for last feedback accuracy and the gap between the current and previous presentation of the item pair. Participants are treated as random effects with respect to the intercepts. n=31 participants x20 item pairs per trial. "Trial number" indicates the number of times the item pair has been presented. "Last feedback" indicates whether the feedback presented on the previous presentation of the item pair was accurate (=1) or not (=0). "Gap" is the number of trials between the current and previous presentation of the item pair. "Δv" is the participant's bid for the correct item minus the participant's bid for the incorrect item. * P<0.05, ** P<0.01, *** P<0.001.

	Variable	Coefficient (SE)	*t* value
**Social, trials 2-30**			
LL without Δv: -23283	(Intercept)	0.581 (0.021)	28.1
LL with Δv: -23198***	Trial number	-0.037 (0.001)	-46.9
$\chi^2(1)$=169.78	Last feedback	-0.102 (0.016)	-6.2
	Gap	0.003 (0.0004)	8.0
	Δv	-0.120 (0.009)	-13.1
**Social, trials 2-10**			
LL without Δv: -8650	(Intercept)	1.259 (0.057)	22.0
LL with Δv: -8606***	Trial number	-0.127 (0.006)	-21.8
$\chi^2(1)$=87.41	Last feedback	-0.215 (0.038)	-5.7
	Gap	0.007 (0.001)	6.7
	Δv	-0.202 (0.021)	-9.4
**Social, trials 11-20**			
LL without Δv: -7170	(Intercept)	0.157 (0.061)	2.6
LL with Δv: -7145***	Trial number	-0.022 (0.003)	-6.5
$\chi^2(1)$=51.26	Last feedback	-0.077 (0.024)	-3.2
	Gap	0.004 (0.001)	6.3
	Δv	-0.101 (0.014)	-7.2
**Social, trials 21-30**			
LL without Δv: -5761	(Intercept)	-0.149 (0.076)	-2.0
LL with Δv: -5730***	Trial number	-0.006 (0.003)	-2.3
$\chi^2(1)$=61.91	Last feedback	-0.023 (0.020)	-1.1
	Gap	0.002 (0.0005)	3.6
	Δv	-0.090 (0.011)	-7.9

Supplementary Table 6. Response times in the social experiment, z-scored by participant, with additional controls. Models are mixed-effects linear regressions using bids as the preference congruence measure and controlling for last feedback accuracy and the gap between the current and previous presentation of the item pair. Participants are treated as random effects with respect to the intercepts. Across 31 participants, trials 2-30, *n*=17,976 observations; trials 2-10, *n*=5,579 observations; trials 11-20, *n*=6,198 observations; trials 21-30, *n*=6,199 observations; response times for four trials were excluded *a priori* due to recording errors. "Trial number" indicates the number of times the item pair has been presented. "Last feedback" indicates whether the feedback presented on the previous presentation of the item pair was accurate (=1) or not (=0). "Gap" is the number of trials between the current and previous presentation of the item pair. "Δv" is the participant's bid for the item matching her response minus her bid for its alternative. *P* values are not noted for coefficients due to ambiguities in their interpretation for mixed-effects linear models. Due to this ambiguity, effects of Δv were evaluated based on whether its inclusion significantly improved the model's fit as measured by a χ^2 test of their log-likelihoods (LL). * P <0.05, ** P <0.01, *** P <0.001.

	Variable	Coefficient (SE)	*z* value
**Social, trials 2-30**			
	(Intercept)	0.657*** (0.139)	4.7
	Trial number	0.093*** (0.004)	23.5
	Last feedback	0.985*** (0.063)	15.7
	Gap	-0.003 (0.002)	-1.5
	Same choice	0.429*** (0.031)	13.8
**Social, trials 2-10**			
	(Intercept)	-0.139 (0.147)	-0.9
	Trial number	0.173*** (0.016)	10.7
	Last feedback	1.416*** (0.085)	16.6
	Gap	-0.004 (0.003)	-1.6
	Same choice	0.458*** (0.042)	10.8
**Social, trials 11-20**			
	(Intercept)	1.236** (0.382)	3.2
	Trial number	0.107*** (0.020)	5.4
	Last feedback	0.493*** (0.125)	4.0
	Gap	-0.004 (0.003)	-1.1
	Same choice	0.360*** (0.058)	6.2
**Social, trials 21-30**			
	(Intercept)	3.050*** (0.691)	4.4
	Trial number	0.022 (0.024)	0.9
	Last feedback	0.403* (0.159)	2.5
	Gap	0.001 (0.004)	0.2
	Same choice	0.495*** (0.074)	6.7

Supplementary Table 7. Correct responses in the social experiment with additional controls. Models are mixed-effects logistic regressions using choices as the preference congruence measure and controlling for last feedback accuracy and the gap between the current and previous presentation of the item pair. Participants are treated as random effects with respect to the intercepts. *n*=31 participants x 20 item pairs per trial. "Trial number" indicates the number of times the item pair has been presented. "Last feedback" indicates whether the feedback presented on the previous presentation of the item pair was accurate (=1) or not (=0). "Gap" is the number of trials between the current and previous presentation of the item pair. "Same choice" is whether the participant consistently chose the same item as her partner (same=1, other=-1); because participants indicated choices between each item pair twice, inconsistent choices were coded as 0. * $P<0.05$, ** $P<0.01$, *** $P<0.001$.

	Variable	Coefficient (SE)	*t* value
**Social, trials 2-30**			
LL without choice: -23283	(Intercept)	0.582 (0.021)	28.1
LL with choice: -23211***	Trial number	-0.037 (0.001)	-47.1
$\chi^2(1)$=144.51	Last feedback	-0.101 (0.016)	-6.2
	Gap	0.003 (0.0004)	8.0
	Same choice	-0.083 (0.007)	-12.0
**Social, trials 2-10**			
LL without choice: -8650	(Intercept)	1.259 (0.057)	22.0
LL with choice: -8607***	Trial number	-0.128 (0.006)	-21.8
$\chi^2(1)$=84.83	Last feedback	-0.210 (0.038)	-5.6
	Gap	0.006 (0.001)	6.6
	Same choice	-0.150 (0.016)	-9.2
**Social, trials 11-20**			
LL without choice: -7170	(Intercept)	0.154 (0.061)	2.5
LL with choice: -7156***	Trial number	-0.023 (0.003)	-6.6
$\chi^2(1)$=28.77	Last feedback	-0.075 (0.024)	-3.1
	Gap	0.004 (0.001)	6.4
	Same choice	-0.056 (0.010)	-5.4
**Social, trials 21-30**			
LL without choice: -5761	(Intercept)	-0.148 (0.076)	-1.9
LL with choice: -5735***	Trial number	-0.006 (0.003)	-2.3
$\chi^2(1)$=51.31	Last feedback	-0.025 (0.020)	-1.3
	Gap	0.002 (0.0005)	3.6
	Same choice	-0.060 (0.008)	-7.2

Supplementary Table 8. Response times in the social experiment, z-scored by participant, with additional controls. Models are mixed-effects linear regressions using choices as the preference congruence measure and controlling for last feedback accuracy and the gap between the current and previous presentation of the item pair. Participants are treated as random effects with respect to the intercepts. Across 31 participants, trials 2-30, *n*=17,976 observations; trials 2-10, *n*=5,579 observations; trials 11-20, *n*=6,198 observations; trials 21-30, *n*=6,199 observations; response times for four trials were excluded *a priori* due to recording errors. "Trial number" indicates the number of times the item pair has been presented. "Last feedback" indicates whether the feedback presented on the previous presentation of the item pair was accurate (=1) or not (=0). "Gap" is the number of trials between the current and previous presentation of the item pair. "Same choice" is whether the participant's response matched her preference during the choice task (matched=1, did not match=-1); because participants indicated choices between each item pair twice, inconsistent choices were coded as 0. *P* values are not noted for coefficients due to ambiguities in their interpretation for mixed-effects linear models. Due to this ambiguity, effects of the "same choice" variable were evaluated based on whether its inclusion significantly improved the model's fit as measured by a χ^2 test of their log-likelihoods (LL). * P <0.05, ** P <0.01, *** P <0.001.

	Variable	Coefficient (SE)	*z* value
**Non-social, trials 1-30**			
	(Intercept)	0.760*** (0.107)	7.1
	Trial number	0.103*** (0.003)	31.8
	Δv	0.439*** (0.033)	13.1
**Non-social, trials 1-10**			
	(Intercept)	0.113 (0.102)	1.1
	Trial number	0.230*** (0.012)	18.8
	Δv	0.585*** (0.047)	12.5
**Non-social, trials 11-20**			
	(Intercept)	1.422*** (0.318)	4.5
	Trial number	0.087*** (0.017)	5.1
	Δv	0.281*** (0.064)	4.4
**Non-social, trials 21-30**			
	(Intercept)	3.047*** (0.605)	5.0
	Trial number	0.024 (0.021)	1.2
	Δv	0.290*** (0.074)	3.9

Supplementary Table 9. Correct responses in the non-social experiment. Models are mixed-effects logistic regressions using bids as the preference congruence measure. Participants are treated as random effects with respect to the intercepts. n=30 participants x 20 item pairs per trial. "Trial number" indicates the number of times the item pair has been presented. "*Δv*" is the participant's bid for the correct item minus the participant's bid for the incorrect item. * P<0.05, ** P<0.01, *** P<0.001.

	Variable	Coefficient (SE)	*t* value
**Non-social, trials 1-30**			
LL without *Δv*: -24482	(Intercept)	0.610 (0.014)	42.2
LL with *Δv*: -24444***	Trial number	-0.039 (0.001)	-47.7
$\chi^2(1)$=74.62	*Δv*	-0.080 (0.009)	-8.7
**Non-social, trials 1-10**			
LL without *Δv*: -9168	(Intercept)	1.118 (0.052)	21.4
LL with *Δv*: -9138***	Trial number	-0.118 (0.005)	-23.8
$\chi^2(1)$=58.92	*Δv*	-0.149 (0.019)	-7.7
**Non-social, trials 11-20**			
LL without *Δv*: -7191	(Intercept)	0.083 (0.059)	1.4
LL with *Δv*: -7175***	Trial number	-0.014 (0.004)	-3.9
$\chi^2(1)$=30.45	*Δv*	-0.077 (0.014)	-5.5
**Non-social, trials 21-30**			
LL without *Δv*: -7133	(Intercept)	0.066 (0.096)	0.7
LL with *Δv*: -7127***	Trial number	-0.014 (0.004)	-4.0
$\chi^2(1)$=12.10	*Δv*	-0.048 (0.014)	-3.5

Supplementary Table 10. Response times in the non-social experiment, z-scored by participant. Models are mixed-effects linear regressions using bids as the preference congruence measure. Participants are treated as random effects with respect to the intercepts. Across 30 participants, trials 1-30, n=17,999 observations; trials 1-10, n=6,000 observations; trials 11-20, n=5,999 observations; trials 21-30, n=6,000 observations; the response time for one trial was excluded *a priori* due to a recording error. "Trial number" indicates the number of times the item pair has been presented. "*Δv*" is the participant's bid for the item matching her response minus her bid for its alternative. P values are not noted for coefficients due to ambiguities in their interpretation for mixed-effects linear models. Due to this ambiguity, effects of *Δv* were evaluated based on whether its inclusion significantly improved the model's fit as measured by a χ^2 test of their log-likelihoods (LL). * P<0.05, ** P<0.01, *** P<0.001.

	Variable	Coefficient (SE)	*z* value
**Non-social, trials 1-30**			
	(Intercept)	0.772*** (0.109)	7.1
	Trial number	0.105*** (0.003)	31.9
	Same choice	0.509*** (0.027)	19.2
**Non-social, trials 1-10**			
	(Intercept)	0.112 (0.104)	1.1
	Trial number	0.233*** (0.012)	18.9
	Same choice	0.538*** (0.035)	15.2
**Non-social, trials 11-20**			
	(Intercept)	1.449*** (0.321)	4.5
	Trial number	0.089*** (0.017)	5.2
	Same choice	0.539*** (0.054)	10.0
**Non-social, trials 21-30**			
	(Intercept)	3.084*** (0.609)	5.1
	Trial number	0.024 (0.021)	1.2
	Same choice	0.471*** (0.065)	7.2

Supplementary Table 11. Correct responses in the non-social experiment. Models are mixed-effects logistic regressions using choices as the preference congruence measure. Participants are treated as random effects with respect to the intercepts. n=30 participants x 20 item pairs per trial. "Same choice" is whether the participant consistently chose the same item as her partner (same=1, other=-1); because participants indicated choices between each item pair twice, inconsistent choices were coded as 0. * P<0.05, ** P<0.01, *** P<0.001.

	Variable	Coefficient (SE)	*t* value
**Non-social, trials 1-30**			
LL without choice: -24482	(Intercept)	0.617 (0.014)	42.6
LL with choice: -24418***	Trial number	-0.039 (0.001)	-47.9
$\chi^2(1)$=126.29	Same choice	-0.082 (0.007)	-11.3
**Non-social, trials 1-10**			
LL without choice: -9168	(Intercept)	1.132 (0.052)	21.7
LL with choice: -9124***	Trial number	-0.119 (0.005)	-24.0
$\chi^2(1)$=88.07	Same choice	-0.144 (0.015)	-9.4
**Non-social, trials 11-20**			
LL without choice: -7191	(Intercept)	0.084 (0.059)	1.4
LL with choice: -7168***	Trial number	-0.014 (0.004)	-3.9
$\chi^2(1)$=44.98	Same choice	-0.074 (0.011)	-6.7
**Non-social, trials 21-30**			
LL without choice: -7133	(Intercept)	0.072 (0.096)	0.7
LL with choice: -7118***	Trial number	-0.014 (0.004)	-4.0
$\chi^2(1)$=29.24	Same choice	-0.059 (0.011)	-5.4

Supplementary Table 12. Response times in the non-social experiment, z-scored by participant. Models are mixed-effects linear regressions using choices as the preference congruence measure. Participants are treated as random effects with respect to the intercepts. Across 30 participants, trials 1-30, n=17,999 observations; trials 1-10, n=6,000 observations; trials 11-20, n=5,999 observations; trials 21-30, n=6,000 observations; the response time for one trial was excluded *a priori* due to a recording error. "Trial number" indicates the number of times the item pair has been presented. "Same choice" is whether the participant's response matched her preference during the choice task (matched=1, did not match=-1); because participants indicated choices between each item pair twice, inconsistent choices were coded as 0. P values are not noted for coefficients due to ambiguities in their interpretation for mixed-effects linear models. Due to this ambiguity, effects of the "same choice" variable were evaluated based on whether its inclusion significantly improved the model's fit as measured by a χ^2 test of their log-likelihoods (LL). * P<0.05, ** P<0.01, *** P<0.001.

	Variable	Coefficient (SE)	*z* value
**Non-social, trials 2-30**			
	(Intercept)	0.336* (0.132)	2.5
	Trial number	0.093*** (0.003)	26.9
	Last feedback	0.926*** (0.057)	16.3
	Gap	-0.004* (0.002)	-2.4
	Δv	0.385*** (0.035)	10.9
**Non-social, trials 2-10**			
	(Intercept)	-0.586*** (0.150)	-3.9
	Trial number	0.207*** (0.015)	13.9
	Last feedback	1.346*** (0.082)	16.4
	Gap	-0.007** (0.002)	-3.2
	Δv	0.499*** (0.052)	9.6
**Non-social, trials 11-20**			
	(Intercept)	1.025** (0.342)	3.0
	Trial number	0.086*** (0.017)	5.0
	Last feedback	0.583*** (0.111)	5.3
	Gap	-0.002 (0.003)	-0.6
	Δv	0.287*** (0.065)	4.4
**Non-social, trials 21-30**			
	(Intercept)	2.842*** (0.632)	4.5
	Trial number	0.021 (0.021)	1.0
	Last feedback	0.418** (0.135)	3.1
	Gap	-0.003 (0.003)	-0.8
	Δv	0.293*** (0.074)	3.9

Supplementary Table 13. Correct responses in the non-social experiment with additional controls. Models are mixed-effects logistic regressions using bids as the preference congruence measure and controlling for last feedback accuracy and the gap between the current and previous presentation of the item pair. Participants are treated as random effects with respect to the intercepts. n=30 participants x20 item pairs per trial. "Trial number" indicates the number of times the item pair has been presented. "Last feedback" indicates whether the feedback presented on the previous presentation of the item pair was accurate (=1) or not (=0). "Gap" is the number of trials between the current and previous presentation of the item pair. "Δv" is the participant's bid for the correct item minus the participant's bid for the incorrect item. * P<0.05, ** P<0.01, *** P<0.001.

	Variable	Coefficient (SE)	*t* value
**Non-social, trials 2-30**			
LL without Δv: -23410	(Intercept)	0.549 (0.022)	24.6
LL with Δv: -23372***	Trial number	-0.036 (0.001)	-42.9
$\chi^2(1)$=75.10	Last feedback	-0.070 (0.018)	-3.9
	Gap	0.003 (0.0004)	8.0
	Δv	-0.081 (0.009)	-8.7
**Non-social, trials 2-10**			
LL without Δv: -8204	(Intercept)	1.084 (0.063)	17.2
LL with Δv: -8178***	Trial number	-0.122 (0.006)	-21.1
$\chi^2(1)$=50.89	Last feedback	-0.119 (0.038)	-3.1
	Gap	0.009 (0.001)	9.2
	Δv	-0.144 (0.020)	-7.2
**Non-social, trials 11-20**			
LL without Δv: -7179	(Intercept)	0.045 (0.065)	0.7
LL with Δv: -7164***	Trial number	-0.012 (0.004)	-3.2
$\chi^2(1)$=30.88	Last feedback	-0.059 (0.026)	-2.2
	Gap	0.003 (0.001)	4.4
	Δv	-0.077 (0.014)	-5.6
**Non-social, trials 21-30**			
LL without Δv: -7128	(Intercept)	0.006 (0.101)	0.1
LL with Δv: -7122***	Trial number	-0.013 (0.004)	-3.5
$\chi^2(1)$=12.32	Last feedback	-0.015 (0.025)	-0.6
	Gap	0.002 (0.001)	2.9
	Δv	-0.048 (0.014)	-3.5

Supplementary Table 14. Response times in the non-social experiment, z-scored by participant, with additional controls. Models are mixed-effects linear regressions using bids as the preference congruence measure and controlling for last feedback accuracy and the gap between the current and previous presentation of the item pair. Participants are treated as random effects with respect to the intercepts. Across 30 participants, trials 2-30, *n*=17,399 observations; trials 2-10, *n*=5,400 observations; trials 11-20, *n*=5,999 observations; trials 21-30, *n*=6,000 observations; the response time for one trial was excluded *a priori* due to a recording error. "Trial number" indicates the number of times the item pair has been presented. "Last feedback" indicates whether the feedback presented on the previous presentation of the item pair was accurate (=1) or not (=0). "Gap" is the number of trials between the current and previous presentation of the item pair. "Δv" is the participant's bid for the item matching her response minus her bid for its alternative. *P* values are not noted for coefficients due to ambiguities in their interpretation for mixed-effects linear models. Due to this ambiguity, effects of Δv were evaluated based on whether its inclusion significantly improved the model's fit as measured by a χ^2 test of their log-likelihoods (LL). * P<0.05, ** P<0.01, *** P<0.001.

	Variable	Coefficient (SE)	*z* value
**Non-social, trials 2-30**			
	(Intercept)	0.351** (0.134)	2.6
	Trial number	0.094*** (0.003)	27.0
	Last feedback	0.926*** (0.057)	16.1
	Gap	-0.004* (0.002)	-2.5
	Same choice	0.463*** (0.028)	16.4
**Non-social, trials 2-10**			
	(Intercept)	-0.581*** (0.152)	-3.8
	Trial number	0.210*** (0.015)	14.0
	Last feedback	1.340*** (0.082)	16.3
	Gap	-0.008** (0.002)	-3.2
	Same choice	0.447*** (0.040)	11.3
**Non-social, trials 11-20**			
	(Intercept)	1.061** (0.345)	3.1
	Trial number	0.087*** (0.017)	5.0
	Last feedback	0.584*** (0.112)	5.2
	Gap	-0.002 (0.003)	-0.6
	Same choice	0.541*** (0.054)	10.0
**Non-social, trials 21-30**			
	(Intercept)	2.878*** (0.636)	4.5
	Trial number	0.022 (0.021)	1.0
	Last feedback	0.431** (0.136)	3.2
	Gap	-0.003 (0.004)	-0.9
	Same choice	0.476*** (0.066)	7.2

Supplementary Table 15. Correct responses in the non-social experiment with additional controls. Models are mixed-effects logistic regressions using choices as the preference congruence measure and controlling for last feedback accuracy and the gap between the current and previous presentation of the item pair. Participants are treated as random effects with respect to the intercepts. n=30 participants x 20 item pairs per trial. "Trial number" indicates the number of times the item pair has been presented. "Last feedback" indicates whether the feedback presented on the previous presentation of the item pair was accurate (=1) or not (=0). "Gap" is the number of trials between the current and previous presentation of the item pair. "Same choice" is whether the participant consistently chose the same item as her partner (same=1, other=-1); because participants indicated choices between each item pair twice, inconsistent choices were coded as 0. * P<0.05, ** P<0.01, *** P<0.001.

	Variable	Coefficient (SE)	*t* value
**Non-social, trials 2-30**			
LL without choice: -23410	(Intercept)	0.553 (0.022)	24.8
LL with choice: -23348***	Trial number	-0.036 (0.001)	-43.0
$\chi^2(1)$=123.82	Last feedback	-0.069 (0.018)	-3.9
	Gap	0.003 (0.0004)	8.0
	Same choice	-0.081 (0.007)	-11.2
**Non-social, trials 2-10**			
LL without choice: -8204	(Intercept)	1.089 (0.063)	17.3
LL with choice: -8169***	Trial number	-0.122 (0.006)	-21.2
$\chi^2(1)$=70.49	Last feedback	-0.118 (0.038)	-3.1
	Gap	0.009 (0.001)	9.2
	Same choice	-0.134 (0.016)	-8.4
**Non-social, trials 11-20**			
LL without choice: -7179	(Intercept)	0.045 (0.065)	0.7
LL with choice: -7156***	Trial number	-0.011 (0.004)	-3.2
$\chi^2(1)$=45.30	Last feedback	-0.058 (0.026)	-2.2
	Gap	0.003 (0.001)	4.4
	Same choice	-0.074 (0.011)	-6.8
**Non-social, trials 21-30**			
LL without choice: -7128	(Intercept)	0.011 (0.101)	0.1
LL with choice: -7114***	Trial number	-0.013 (0.004)	-3.6
$\chi^2(1)$=29.68	Last feedback	-0.015 (0.025)	-0.6
	Gap	0.002 (0.001)	2.9
	Same choice	-0.059 (0.011)	-5.5

Supplementary Table 16. Response times in the non-social experiment, z-scored by participant, with additional controls. Models are mixed-effects linear regressions using choices as the preference congruence measure and controlling for last feedback accuracy and the gap between the current and previous presentation of the item pair. Participants are treated as random effects with respect to the intercepts. Across 30 participants, trials 2-30, n=17,399 observations; trials 2-10, n=5,400 observations; trials 11-20, n=5,999 observations; trials 21-30, n=6,000 observations; the response time for one trial was excluded *a priori* due to a recording error. “Trial number” indicates the number of times the item pair has been presented. “Last feedback” indicates whether the feedback presented on the previous presentation of the item pair was accurate (=1) or not (=0). “Gap” is the number of trials between the current and previous presentation of the item pair. “Same choice” is whether the participant’s response matched her preference during the choice task (matched=1, did not match=-1); because participants indicated choices between each item pair twice, inconsistent choices were coded as 0. *P* values are not noted for coefficients due to ambiguities in their interpretation for mixed-effects linear models. Due to this ambiguity, effects of the “same choice” variable were evaluated based on whether its inclusion significantly improved the model’s fit as measured by a χ^2 test of their log-likelihoods (LL). * P<0.05, ** P<0.01, *** P<0.001.

	Variable	Coefficient (SE)	*z*/*t* value
**Last feedback (social)**			
	(Intercept)	1.396*** (0.019)	z=72.0
	Δv	0.009 (0.026)	z=0.3
**Gap (social)**			
	(Intercept)	18.517*** (0.125)	t=147.6
	Δv	0.063 (0.169)	t=0.4
**Last feedback (non-social)**			
	(Intercept)	1.418*** (0.019)	z=73.4
	Δv	-0.0002 (0.025)	z=-0.01
**Gap (non-social)**			
	(Intercept)	18.520*** (0.127)	t=146.4
	Δv	0.053 (0.165)	t=0.3

Supplementary Table 17. Regression models confirming that preference congruence predicts neither the accuracy of previous feedback presented for an item pair or the distance between subsequent presentations of item pairs. Social experiment, n=17,980 observations across 31 participants; non-social experiment, n=17,400 observations across 30 participants. "Last feedback" indicates whether the feedback presented on the previous presentation of the item pair was accurate (=1) or not (=0). "Gap" is the number of trials between the current and previous presentation of the item pair. "Δv" is the participant's bid for the correct item minus the participant's bid for incorrect item.
* P<0.05, ** P<0.01, *** P<0.001.

	Variable	Coefficient (SE)	*z* value
**Social & non-social**			
	(Intercept)	-0.001 (0.092)	-0.01
	Δv	1.545*** (0.116)	13.4
	Social group	0.381** (0.130)	2.9
**Social & non-social with group x Δv interaction**			
	(Intercept)	0.005 (0.092)	0.1
	Δv	1.463*** (0.156)	9.4
	Social group	0.371** (0.131)	2.8
	Δv x Social group	0.175 (0.232)	0.8
**Social with item popularity**			
LL without popularity: -318	(Intercept)	-1.157*** (0.270)	-4.3
LL with popularity: -298***	Δv	1.175*** (0.181)	6.5
$\chi^2(1)=38.26$	Popularity	2.827*** (0.469)	6.0
**Non-social with item popularity**			
LL without popularity: -329	(Intercept)	-0.749** (0.278)	-2.7
LL with popularity: -325**	Δv	1.257*** (0.168)	7.5
$\chi^2(1)=8.1$	Popularity	1.303** (0.459)	2.8
**Social & non-social with item popularity & group x popularity interaction**			
LL without interaction: -669	(Intercept)	-0.852** (0.263)	-3.2
LL with interaction: -667*	Δv	1.196*** (0.122)	9.8
$\chi^2(1)=5.7$	Social group	-0.360 (0.360)	-1.0
	Popularity	1.511*** (0.427)	3.5
	Social group x Popularity	1.413* (0.594)	2.4

Supplementary Table 18. Correct responses on the first trial of each item pair in the social and non-social experiments. The models are mixed-effects logistic regressions using bids as the preference congruence measure. Participants are treated as random effects with respect to the intercept. Social group, *n*=620 observations across 31 participants; non-social group, *n*=600 observations across 30 participants; social and non-social groups combined, *n*=1,220 observations across 61 participants. "Δv" is the participant's bid for the correct item minus the participant's bid for the incorrect item. "Social group" indicates whether the participant was in the social (=1) or non-social (=0) group. "Popularity" is the fraction (between 0 and 1) of the 60 other participants who bid more for the correct item than the incorrect item. * $P<0.05$, ** $P<0.01$, *** $P<0.001$.

Due to the strong correlation between the participant's preference for an item (Δv) and that item's popularity with other participants ($r=0.47$ in the social group, $r=0.49$ in the non-social group, each $P<1.0\times10^{-34}$), it is possible that this popularity variable simply captures some variance in the participant's own latent preference that is not represented by the Δv measure. However, because these correlations do not differ significantly between the social and non-social groups (Fisher's $z=0.36$, $P=0.72$), they would not explain an item's popularity having a greater effect for the social group than for the non-social group.

Item A	Item B	Number prefer A / B / Neither<br>Mean bid for A / B
Bounty Bar	Canned Sweetcorn	30 / 29 / 2<br>£0.75 / £0.82
McCoy's Flame Grilled Steak Potato Chips	Ella's Kitchen Strawberry & Apple Purée	35 / 18 / 8<br>£0.81 / £0.60
Peanut M&Ms	Mr. Porky Pork Crackles	50 / 7 / 4<br>£1.31 / £0.54
Dairy Milk Caramel Bar	Heinz Spaghetti Hoops	47 / 9 / 5<br>£0.86 / 0.47
Aunty's Steamed Puddings	Fruit Pastilles	38 / 19 / 4<br>£0.89 / £0.63
Nakd Banana Crunch Bar	Treacle & Oat Cookies	13 / 47 / 1<br>£0.81 / £1.38
Daim Bar	Olives	29 / 30 / 2<br>£0.84 / £0.84
Crunchie Bar	Rice Pudding	38 / 17 / 6<br>£1.08 / £0.59
Hartley's Raspberries in Jelly	Mars Bar	12 / 43 / 6<br>£0.56 / £0.88
Hobnobs Cookies	Kit Kat Bar	49 / 12 / 0<br>£1.38 / £0.95
Raisins	Yorkie Bar	30 / 28 / 3<br>£0.79 / £0.77
Fig Rolls	Hula Hoops	35 / 21 / 5<br>£0.85 / £0.67
Lemon Cake Slices	Skips Prawn Cocktail Crisps	44 / 14 / 3<br>£1.07 / £0.71
Crunch Bar	Walkers Smoky Bacon Potato Chips	47 / 12 / 2<br>£1.08 / £0.73
Chewy Nougat	Peanuts	12 / 46 / 3<br>£0.56 / £1.03
Pistachios	Heinz Beans	52 / 7 / 2<br>£1.41 / £0.65
Twix Bar	Polo Fruits Candies	55 / 5 / 1<br>£0.93 / £0.49
Canned Pineapple	Sardines	38 / 17 / 6<br>£0.73 / £0.49
Sour Patch Kids	Maltesers	9 / 49 / 3<br>£0.79 / £1.27
Liquorice Catherine Wheels	Jacob's Mini Cheddars	8 / 48 / 5<br>£0.42 / £0.96

Supplementary Table 19. Item pairs and preferences for participants in the social and non-social experiments (*n*=61). Preferences are measured by whether a participant bid more for an item than its alternative. "Neither" means a participant bid the same amount for both items.

	Variable	Coefficient (SE)	*t* value
**Social & non-social, first trials**			
LL without group: -1999	(Intercept)	0.998 (0.105)	9.5
LL with group: -1990***	*Δv*	-0.297 (0.054)	-5.5
$\chi^2(1)$=17.6	Social group	0.648 (0.146)	4.4
**Social & non-social, all other trials**			
LL without group: -48593	(Intercept)	-0.019 (0.007)	-2.5
LL with group: -48592	*Δv*	-0.099 (0.007)	-14.3
$\chi^2(1)$=2.1	Social group	-0.015 (0.010)	-1.4

Supplementary Table 20. Response times on the first trial of each item pair, z-scored by participant. Models are mixed-effects linear regressions using bids as the preference congruence measure. Participants are treated as random effects with respect to the intercept. First trials, *n*=1,220 observations across 61 participants; other trials, *n*=35,375 observations across 61 participants; response times for five trials were excluded *a priori* due to recording errors. "*Δv*" is the participant's bid for the item matching her response minus her bid for its alternative. "Social group" indicates whether the participant was in the social (=1) or non-social (=0) group. *P* values are not noted for coefficients due to ambiguities in their interpretation for mixed-effects linear models. Due to this ambiguity, effects of the "social group" variable were evaluated based on whether its inclusion significantly improved the model's fit as measured by a χ^2 test of their log-likelihoods. * *P*<0.05, ** *P*<0.01, *** *P*<0.001.

	Percentile	Data	Model
**Social, all trials**			
	25th	0.77 s	0.76 s
	50th	0.93 s	0.95 s
	75th	1.23 s	1.25 s
**Social, response = preference (*Δv*>0)**			
	25th	0.75 s	0.75 s
	50th	0.90 s	0.92 s
	75th	1.18 s	1.22 s
**Social, response ≠ preference (*Δv*<0)**			
	25th	0.80 s	0.79 s
	50th	0.98 s	0.99 s
	75th	1.32 s	1.31 s
**Non-social, all trials**			
	25th	0.80 s	0.78 s
	50th	0.98 s	0.99 s
	75th	1.32 s	1.34 s
**Non-social, response = preference (*Δv*>0)**			
	25th	0.77 s	0.77 s
	50th	0.95 s	0.97 s
	75th	1.27 s	1.33 s
**Social, response ≠ preference (*Δv*<0)**			
	25th	0.83 s	0.80 s
	50th	1.02 s	1.01 s
	75th	1.37 s	1.36 s

Supplementary Table 21. Response time quartiles in the data and predicted by the dual influence model with item popularity. Social, all trials, *n*=18,596 observations across 31 participants; social, response matches preference, *n*=10,962; social, response does not match preference, *n*=6,524; non-social, all trials, *n*=17,999 observations across 30 participants; non-social, response matches preference, *n*=9,868; non-social, response does not match preference, *n*=7,111. *Δv*=0 for 1,110 trials in the social group and 1,020 trials in the non-social group and are therefore only included in the "all trials" data. "*Δv*" is the participant's bid for the item matching her response minus her bid for its alternative.

	Comparison	Z
**Social**		
	Mean (trial 1) – Mean (trial 2) = -0.18	-12.7***
**Non-social**		
	Mean (trial 1) – Mean (trial 2) = -0.11	-7.2***

Supplementary Table 22. Permutation tests of the difference in mean probabilities, between the first and second trials of each item pair, of the observed response given the dual influence model with item popularity. * P<0.05, ** P<0.01, *** P<0.001.

	Variable	Coefficient (SE)	*z* value
**Social pilot, trials 1-30**			
	(Intercept)	0.944*** (0.159)	5.9
	Trial number	0.070*** (0.005)	15.5
	Δv	0.282*** (0.039)	7.2
**Social pilot, trials 1-10**			
	(Intercept)	0.155 (0.103)	1.5
	Trial number	0.208*** (0.019)	10.9
	Δv	0.29*** (0.058)	5.0
**Social pilot, trials 11-20**			
	(Intercept)	1.382** (0.482)	2.9
	Trial number	0.076** (0.026)	2.9
	Δv	0.237** (0.078)	3.0
**Social pilot, trials 21-30**			
	(Intercept)	2.477** (0.817)	3.0
	Trial number	0.018 (0.027)	0.7
	Δv	0.326*** (0.075)	4.3

Supplementary Table 23. Correct responses in the social pilot experiment. Models are mixed-effects logistic regressions using bids as the preference congruence measure. Participants are treated as random effects with respect to the intercepts. n=11 participants x 20 item pairs per trial. "Trial number" indicates the number of times the item pair has been presented. "Δv" is the participant's bid for the correct item minus the participant's bid for the incorrect item. * P<0.05, ** P<0.01, *** P<0.001.

**Parameter description**	**Prior**	**Constraint**
Threshold (group mean)	$\mu_a \approx \mathcal{N}(1, 10)$	(0, 3)
Threshold (group SD)	$\sigma_a \approx \mathcal{U}(0, 1)$	(0, 1)
Threshold (subject-level)	$a \approx \mathcal{G}\left(\left(\frac{\mu_a}{\sigma_a}\right)^2, \frac{\mu_a}{{\sigma_a}^2}\right)$	(0, ∞)
Drift weight (group mean)	$\mu_\omega \approx \mathcal{N}(0, 20)$	(-3, 10)
Drift weight (group SD)	$\sigma_\omega \approx half\ Cauchy(0, 0.5)$	(0, 0.5tan(π/2))
Drift weight (subject-level)	$\omega \approx \mathcal{N}(\mu_\omega, \sigma_\omega)$	(-∞,∞)
Inverse temperature of Δv's effect on prior (group mean)	$\mu_{\beta_{\Delta v}} \approx \mathcal{N}(0, 5)$	(-5, 5)
Inverse temperature of Δv's effect on prior (group SD)	$\sigma_{\beta_{\Delta v}} \approx half\ Cauchy(0, 2.5)$	(0, 2.5tan(π/2))
Inverse temperature of Δv's effect on prior (subject-level)	$\beta_{\Delta v} \approx \mathcal{N}(\mu_{\beta_{\Delta v}}, \sigma_{\beta_{\Delta v}})$	(-∞,∞)
Inverse temperature of Δv's effect on DDM starting point (group mean)	$\mu_\kappa \approx \mathcal{N}(0, 5)$	(-5, 5)
Inverse temperature of Δv's effect on DDM starting point (group SD)	$\sigma_\kappa \approx half\ Cauchy(0, 2.5)$	(0, 2.5tan(π/2))
Inverse temperature of Δv's effect on DDM starting point (subject-level)	$\kappa \approx \mathcal{N}(\mu_\kappa, \sigma_\kappa)$	(-∞,∞)
Inverse temperature of item popularity's effect on prior (group mean)	$\mu_{\beta_\rho} \approx \mathcal{N}(0, 5)$	(-5, 5)
Inverse temperature of item popularity's effect on prior (group SD)	$\sigma_{\beta_\rho} \approx half\ Cauchy(0, 2.5)$	(0, 2.5tan(π/2))
Inverse temperature of item popularity's effect on prior (subject-level)	$\beta_\rho \approx \mathcal{N}(\mu_{\beta_\rho}, \sigma_{\beta_\rho})$	(-∞,∞)
Non-decision time (subject-level)	$s \approx \mathcal{U}(0, min_n(RT))$	$(0, min_n(RT))$

Supplementary Table 24. Priors and constraints for parameters in the dual influence models. The priors specified for this model were the same as those specified for the same parameters in the other Bayesian and Rescorla-Wagner-type models. Parameters were constrained to within reasonable ranges to aid sampling. SD is the standard deviation; "Δv" is the bid for the correct item minus the bid for the incorrect item; $min_n(RT)$ is the minimum plausible response time for participant n.

Supplementary Methods

Participant instructions: Social experiment
Instructions for the social and non-social experiments were modified from those used in [2].

Food Experiment Instructions: Part 1

Thank you for agreeing to participate in this experiment on decision making. You will be compensated £25 for your time. The experiment should take approximately 2 ½ hours.

You should not have eaten for 3 hours before coming to participate in this experiment. This is important for the success of the experiment so please indicate before the experiment begins if for any reason you weren't able to comply with this requirement.

If at any time you no longer wish to continue with the experiment, you are free to leave at any point.

Bidding Task

Your task in the first stage of the experiment is to indicate the price you are willing to pay to buy various snack items. You will be asked to bid on each of these items, from £0-£3. Indicate your bid by sliding the scale left or right using the arrow keys. When the cursor is in the right place, press the **down** arrow key to enter your bid.

The following screenshot provides an example of the bidding task:

[Screenshot of Topic bar with £0-£3 sliding scale beneath it; full instructions with screenshots available on GitHub; see Data Availability, main text]

Think carefully about the price you bid. Each bid should be the maximum price you are prepared to pay to consume each of these items.

Choice Task

In the second task, you will be asked to choose between a series of snacks. These will be the same snacks you bid on previously, but will be presented in pairs. First you will be asked to indicate which item you prefer using the left and right arrow keys on the keyboard in front of you.

You will then be asked to rate how confident you are that you made the best choice. To measure this, we will ask you to indicate how confident you were that you were correct on each trial. This is on a sliding scale of 1-6, with:

Participant instructions: Social experiment (con't)

1 = relatively low confidence

6 = relatively high confidence

It is important that you try to use the full range of the scale from 1-6 and think hard about how confident you are after each decision. We are interested in **relative** confidence within our task. In other words, even if your confidence only fluctuates a small amount during the task, please indicate this using the whole scale.

The scale looks a bit like this:

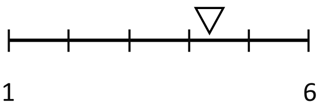

Use the **left** and **right** arrow keys on the keyboard to slide the cursor left and right on the confidence scale. When the cursor is in the right place, press the **down** arrow key to record your answer.

The following 2 screenshots illustrate an example of the choice task:

[Screenshot of Cheestrings and Picnic bar on left, screenshot of confidence slider on right; full instructions with screenshots available on GitHub; see Data Availability, main text]

Here in the first screenshot you're asked to indicate whether you would prefer a bag of Cheestrings to a Picnic bar. In the second, you're asked to provide a rating of confidence. In this example, the participant was reasonably confident of their decision, so they selected a confidence rating just above 5.

Snack Purchase

When you finish, we will select one trial at random from the choice task. We will look at which of the two items you chose and the maximum price you stated you would be happy to pay for that item in the bidding task. In order to establish what the price of the item is and if you buy the item or not, we will run an auction. Let's have a look at how this auction works…

Auction Rules:

We start by looking at your bid for the item (i.e. the maximum price you were happy to pay for it). For example, let's say the item is a Snickers bar and your bid for it in the experiment was £1.64. The "retail" price of the snack (e.g. the Snickers bar) will be randomly generated by computer using a random number generator that can generate a price between £0.01 and

£3.00. Let's say for example that the cost of the Snickers bar generated randomly is established to be

Participant instructions: Social experiment (con't)

£1.13. Since the maximum price you bid (£1.64) is higher than the cost of the item (£1.13), you will buy the item in this instance. However, it is important to realise that you won't pay £1.64 for it—rather, you would pay the randomly generated cost of the Snickers (£1.13). This might seem strange, but think of each price that you bid as the maximum price you are happy to pay, not the

price that you would actually pay, which will be randomly generated. If the randomly generated price were instead established to be £2.17, since this price is higher than your bid of £1.64, you won't purchase the Snickers bar. It's therefore in your interest to state the truthful price you are willing to pay for each item, since this won't affect the cost of the item to you, but it does affect the probability that you buy the item.

Please be aware that you will be required to stay 1 more hour with us after the experiment and the ONLY FOOD you will be allowed to consume during this time will be any item bought during the experiment – we will be very strict about this. So when you are deciding how much to bid for an item, ask yourself how much YOU want that item and how much you are ready to pay for consuming that snack at this time (disregarding how much you would usually expect to pay for each of these items in a grocery store).

This is not such an unusual situation and a real-life example might help to clarify the point. Imagine you go to the cinema and want to buy some popcorn to eat during the film; here it has probably occurred to you at one time or another that the cost of popcorn would be a lot less outside of the cinema (e.g. in a supermarket or at your home) than the prices being demanded by the cinema snack counter. However, if you want to consume popcorn during the film, you have to pay the prices they're proposing and it's up to you to decide if this is a price you are happy paying or not.

At the end of the experiment, we will total your final payment in this way:

  £25 base payment
− cost of item purchased at auction (if an item is purchased)
+ total reward for correct responses in part 2 of the experiment (explained later)
____
= FINAL PAYMENT

You will receive a cheque in the mail for your final payment amount.

Food Experiment Instructions: Part 2

Inferring Another Person's Choices

In the second part of the experiment, you will be asked to figure out which snack choices were made by one other participant in an earlier phase of this study. This person completed the same choice task you just finished, and was asked to choose between the same pairs of items that you were. Your job is to figure out which items he or she chose.

You will be shown several pairs of snacks – the same pairs you saw during the first part of the experiment – and then asked to indicate which item you think the other person chose. Use the left and right arrow keys to make your selection. A red star will appear on the item you selected, as shown in the screen shot below.

[Screenshot of Discos bag on left with red asterisk superimposed and Aero package on right; full instructions with screenshots available on GitHub; see Data Availability, main text]

After making your selection, a yellow box will appear around one of the two items. This is a clue. About 80% of the time, the yellow box will appear around the item that the other person chose. The other 20% of the time, however, it will appear around the item that the person did NOT choose.

[Screenshot of Discos bag on left and Aero package on right surrounded by yellow box; full instructions with screenshots available on GitHub; see Data Availability, main text]

An analogy might help illustrate how this works. Imagine that the person is planning a party and needs to buy 30 bags of discos out of a snack machine that sells only Discos and Aero mousse cups. The snack machine is broken, so although the person chooses Discos each time, the machine dispenses (displays a yellow box around) discos only about 80% of the time and Aero mousse cups the other 20% of the time. It is important to understand that the person's choice never changes, but sometimes the yellow box will appear around different items (because the machine is broken).

You will be shown several different pairs of items many times, in random order. Sometimes the items will appear on different sides of the screen. Although you will see each pair of items several times—and sometimes the items will be flipped on the screen—it's important to understand that the person only made one choice between each pair of items.

To help illustrate, the screen shot on the left, below, shows you the screen that the other person saw when making one of the choices that you will try to figure out. (In this example, the

other person chose the Cheestrings.) The screen shots on the right show you what you will see—several

Participant instructions: Social experiment (con't)

presentations of the same choice (sometimes flipped on the screen), with yellow boxes appearing around the other person's choice (Cheestrings) about 80% of the time, and around the other item (Picnic bar) about 20% of the time. But remember, each of the presentations on the right result from the one single choice shown on the left.

[Screenshots demonstrating the relationship between the other person's choice and the probabilistic feedback; full instructions with screenshots available on GitHub; see Data Availability, main text]

Pay close attention and try to remember each clue that the yellow box gives you – it will help you the next time you see that pair of items. You will earn **1 penny** for each correct response, which will be totalled at the end of the experiment.

Before you start this part of the experiment, we will run some practice trials to make sure you are comfortable with the task and everything is clear. If you have any further questions before the real experiment begins, however, please feel free to ask. It's important that you're clear about these instructions before we start.

Participant instructions: Non-social experiment

Experiment 1: Food Preferences

Thank you for agreeing to participate in this study on decision making. You will be compensated £25 for your time. The entire session should take approximately 2 ½ hours.

You should not have eaten for 3 hours before coming to participate in this study. This is important for the success of the study so please indicate before the study begins if for any reason you weren't able to comply with this requirement.

If at any time you no longer wish to continue with the study, you are free to leave at any point.

Bidding Task

Your task in the first experiment is to indicate the price you are willing to pay to buy various snack items. You will be asked to bid on each of these items, from £0-£3. Indicate your bid by sliding the scale left or right using the arrow keys. When the cursor is in the right place, press the **down** arrow key to enter your bid.

The following screenshot provides an example of the bidding task:

[Screenshot of Topic bar with £0-£3 sliding scale beneath it; full instructions with screenshots available on GitHub; see Data Availability, main text]

Think carefully about the price you bid. Each bid should be the maximum price you are prepared to pay to consume each of these items.

Choice Task

In the second task, you will be asked to choose between a series of snacks. These will be the same snacks you bid on previously, but will be presented in pairs. First you will be asked to indicate which item you prefer using the left and right arrow keys on the keyboard in front of you.

You will then be asked to rate how confident you are that you made the best choice. To measure this, we will ask you to indicate how confident you were that you were correct on each trial. This is on a sliding scale of 1-6, with:

1 = relatively low confidence

6 = relatively high confidence

Participant instructions: Non-social experiment (con't)

It is important that you try to use the full range of the scale from 1-6 and think hard about how confident you are after each decision. We are interested in **relative** confidence within our task. In other words, even if your confidence only fluctuates a small amount during the task, please indicate this using the whole scale.
The scale looks a bit like this:

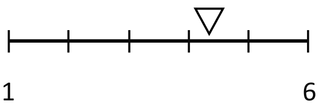

Use the **left** and **right** arrow keys on the keyboard to slide the cursor left and right on the confidence scale. When the cursor is in the right place, press the **down** arrow key to record your answer.

The following 2 screenshots illustrate an example of the choice task:

[Screenshot of Cheestrings and Picnic bar on left, screenshot of confidence slider on right; full instructions with screenshots available on GitHub; see Data Availability, main text]

Here in the first screenshot you're asked to indicate whether you would prefer a bag of Cheestrings to a Picnic bar. In the second, you're asked to provide a rating of confidence. In this example, the participant was reasonably confident of their decision, so they selected a confidence rating just above 5.

Snack Purchase

When you finish, we will select one trial at random from the choice task. We will look at which of the two items you chose and the maximum price you stated you would be happy to pay for that item in the bidding task. In order to establish what the price of the item is and if you buy the item or not, we will run an auction. Let's have a look at how this auction works…

Auction Rules:

We start by looking at your bid for the item (i.e. the maximum price you were happy to pay for it). For example, let's say the item is a Snickers bar and your bid for it in the experiment was £1.64. The "retail" price of the snack (e.g. the Snickers bar) will be randomly generated by computer using a random number generator that can generate a price between £0.01 and £3.00. Let's say for example that the cost of the Snickers bar generated randomly is established to be £1.13. Since the maximum price you bid (£1.64) is higher than the cost of the item (£1.13), you will buy the item in this instance. However, it is important to realise that you won't pay £1.64 for it—rather, you would pay the randomly generated cost of the Snickers (£1.13). This might seem strange, but think of each price that you bid as the maximum price you are

happy to pay, not the price that you would actually pay, which will be randomly generated. If the randomly generated

Participant instructions: Non-social experiment (con't)

price were instead established to be £2.17, since this price is higher than your bid of £1.64, you won't purchase the Snickers bar. It's therefore in your interest to state the truthful price you are willing to pay for each item, since this won't affect the cost of the item to you, but it does affect the probability that you buy the item.

Please be aware that you will be required to stay 1 more hour with us after the experiment and the ONLY FOOD you will be allowed to consume during this time will be any item bought during the experiment – we will be very strict about this. So when you are deciding how much to bid for an item, ask yourself how much YOU want that item and how much you are ready to pay for consuming that snack at this time (disregarding how much you would usually expect to pay for each of these items in a grocery store).

This is not such an unusual situation and a real-life example might help to clarify the point. Imagine you go to the cinema and want to buy some popcorn to eat during the film; here it has probably occurred to you at one time or another that the cost of popcorn would be a lot less outside of the cinema (e.g. in a supermarket or at your home) than the prices being demanded by the cinema snack counter. However, if you want to consume popcorn during the film, you have to pay the prices they're proposing and it's up to you to decide if this is a price you are happy paying or not.

At the end of the experiment, we will total your final payment in this way:

  £25 base payment
− cost of item purchased at auction (if an item is purchased)
+ total reward for correct responses in Experiment 2 (explained later)
____
= FINAL PAYMENT

You will receive a cheque in the mail for your final payment amount.

Experiment 2: Learning Task

In the second experiment, you will be asked to learn a random set of snack items. This set includes one item from each of the pairs you saw in the first experiment.

You will be shown several pairs of snacks – the same pairs you saw during the first experiment – and then asked to indicate which item you think is the item included in the set.

Imagine there are several buckets each filled with 10 snacks. **You can't see** inside the buckets, but on the outside of each one there is a picture of a different pair of snacks. Each bucket contains 8 of one of these snack and 2 of the other, but you don't know which is which. Your goal is to figure out which of the two items the bucket has 8 of.

First, you indicate which item you think is the correct answer. (In this task, you will use the left and right arrow keys to make your selection; a red star will appear on the item you selected, as shown in the screen shot on the bottom left, below.)

[Screenshots illustrating the probabilistic nature of the feedback; full instructions with screenshots available on GitHub; see Data Availability, main text]

After you indicate your answer, one item will be drawn at random from the bucket and then put back for the next trial. In the actual task, a yellow box will appear around **the item that was drawn from the bucket**.

Eventually, based on which item is drawn more often, you'll learn the correct answer. It is important to understand that the correct answer never changes, but sometimes the yellow box will appear around different items (because, in this analogy, each bucket has 2 of the wrong item).

You will be shown several different pairs of items many times, in random order. Sometimes the items will appear on different sides of the screen. Although you will see each pair of items several times—and sometimes the items will be flipped on the screen—it's important to understand that each pair only has one correct answer.

To help illustrate, the screen shots below show you what you might see—several presentations of the same items (sometimes flipped on the screen), with yellow boxes appearing around the correct item (Cheestrings) about 80% of the time, and around the wrong item (Picnic bar) about 20% of the time. But remember, each of these presentations result from one single correct answer (the Cheestrings).

Participant instructions: Non-social experiment (con't)

[Screenshots showing feedback; full instructions with screenshots available on GitHub; see Data Availability, main text]

Pay close attention and try to remember each clue that the yellow box gives you – it will help you the next time you see that pair of items. You will earn **1 penny** for each correct response, which will be totalled at the end of the experiment.

Before you start, we will run some practice trials to make sure you are comfortable with the task and everything is clear. If you have any further questions before the real experiment begins, however, please feel free to ask. It's important that you're clear about these instructions before we start.

Supplementary References

1. Vandekerckhove, J. & Wabersich, D. The RWiener package: An R package providing distribution functions for the Wiener diffusion model. *R J.* **6,** (2014).
2. De Martino, B., Fleming, S. M., Garrett, N. & Dolan, R. J. Confidence in value-based choice. *Nat. Neurosci.* **16,** 105–110 (2012).
